# Metabolomic Profiles of Plasma Retinol-Associated Dyslipidemia in Men and Women

**DOI:** 10.3389/fnut.2021.740435

**Published:** 2021-11-17

**Authors:** Ninglin Wang, Yuan Ru, Zhiying Yang, Changxuan Sun, Shanshan Li, Yan Min, Xueyin Zhao, Ying Lu, Ann W. Hsing, Shankuan Zhu

**Affiliations:** ^1^Chronic Disease Research Institute, The Children's Hospital and National Clinical Research Center for Child Health, School of Public Health, School of Medicine, Zhejiang University, Hangzhou, China; ^2^Department of Nutrition and Food Hygiene, School of Public Health, School of Medicine, Zhejiang University, Hangzhou, China; ^3^Stanford Prevention Research Center, Department of Medicine, Stanford School of Medicine, Stanford University, Stanford, CA, United States; ^4^Department of Biomedical Data Sciences, Stanford School of Medicine, Stanford, CA, United States; ^5^Department of Epidemiology and Population Health, Stanford School of Medicine, Stanford, CA, United States; ^6^Stanford Cancer Institute, School of Medicine, Stanford University, Stanford, CA, United States

**Keywords:** vitamin A, plasma retinol, dyslipidemia, metabolomics profiles, pathway analysis

## Abstract

**Background and Aims:** Studies of both animals and humans show that a high intake of vitamin A is associated with a lower risk of dyslipidemia. However, an association of plasma retinol levels with dyslipidemia is unclear. Therefore, the aim of this study is to investigate an association between plasma retinol and dyslipidemia and to identify related metabolites and pathways in the general population.

**Methods:** We included 250 participants aged 20–80 years from the Wellness Living Laboratory (WELL) China cohort. Associations between plasma retinol levels and dyslipidemia were analyzed using adjusted logistic models. Related metabolites were identified using ANCOVA, adjusted for the false discovery rate (FDR) and used for pathway analyses. Because there are sex differences in plasma retinol levels, all analyses were conducted separately by sex.

**Results:** Plasma retinol was significantly higher in men than in women. A positive association between plasma retinol and dyslipidemia was found in both sexes. In men, the 2nd and 3rd tertiles showed significantly higher proportions of dyslipidemia than the 1st tertile (1st tertile vs. 2nd tertile: *p* = 0.026; 1st tertile vs. 3rd tertile: *p* = 0.003). In women, the 3rd tertile showed a significantly higher proportion of dyslipidemia than the 1st and 2nd tertile (3rd tertile vs. 1st tertile: *p* = 0.002, 3rd tertile vs. 2nd tertile: *p* = 0.002). Overall, 75 and 30 metabolites were significantly associated with retinol levels in men and women, respectively. According to these metabolites, lipid metabolic pathways, including glycerophospholipid, arachidonic acid, linoleic acid, alpha-linolenic acid, and glycosylphosphatidylinositol (GPI), as well as steroid hormone biosynthesis pathways were found to overlap across the sexes. These pathways showed that elevated retinol levels might be associated with hormone metabolism and inflammation status.

**Conclusions:** We found a positive association between plasma retinol levels and dyslipidemia. Related metabolomic profiles and interrupted pathways showed that such an increase might be associated with steroid hormone synthesis and inflammation. In addition, large, population-based longitudinal studies and intervention studies are needed to confirm the role of retinol in lipid metabolism and the prevention of cardiovascular disease (CVD).

## Introduction

Dyslipidemia is a leading risk factor of cardiovascular disease (CVD), which causes ~17.8 million deaths globally in 2017 (1, 2). Dyslipidemia has increased rapidly in China owing to rapid socioeconomic development, and it is now a major contributor to an increase in coronary heart disease mortality (3). According to the National Survey of Chronic Kidney Disease in 2007, the prevalence of dyslipidemia is ~34% in China (4), as compared with 53% in the USA and 20% in Europe (5, 6). While genetics is a cause of dyslipidemia, dietary factors, and nutritional status such as intake of carbohydrates and fatty acids also play important roles (7). Moreover, few studies have shown that micronutrient status, such as vitamins A, C, and D also have a role in lipid metabolism and dyslipidemia (8–10).

Both animal and human population-based studies have shown that higher consumption of vitamin A is associated with lower serum levels of total cholesterol or higher levels of high-density lipoprotein (HDL) cholesterol (11–13). However, as a biomarker of body vitamin A nutritional status, few studies on the role of serum or plasma retinol acting on lipid metabolism are limited (14). Previous evidence shows that high- or low-serum retinol both increase cardiovascular-related mortality (15). A study using a long-term high dosage of retinol (the main type of vitamin A) as dermatosis medication reported that high-serum retinol levels were associated with higher triglycerides and cholesterol and lower HDL cholesterol (16). Another study focused on adolescents showed that participants with dyslipidemia had higher serum concentrations of retinol (17).

Metabolomic assessment, which measures a large number of small-molecule metabolites in the biological system within one assay, has the potential to provide insights into the interrelationships among serum retinol, lipids, and other metabolites. The untargeted metabolomics analysis is an effective way of identifying metabolites that do change in response to the manipulation of a biological system, to better understand metabolic dysregulation (18). Untargeted metabolomic assays can be used to identify related metabolites, such as retinol levels and lipid profiles, and to explain their metabolic pathways. Few studies have reported several metabolic pathways and related metabolites that are associated with dyslipidemia (19).

Therefore, in this study, we aimed to (1) investigate the associations of plasma retinol levels with dyslipidemia; (2) identify the metabolites related to plasma retinol levels; and (3) examine the potential pathways between retinol levels and related metabolites in association with dyslipidemia.

## Methods

### Wellness Living Laboratory China Study and Participants

Wellness Living Laboratory (WELL) China is one of the four cohorts included in the Stanford WELL for Life Global Initiative. For WELL China, we recruited a total of 10,288 participants aged 20–80 years between 2016 and 2019 from a local residential (household) register of residents who were at the same address for more than 6 months, in the three districts of Hangzhou (Xi'hu, Shangcheng, and Gongshu). Eligible participants were physically and mentally capable of participating in the baseline information collection. Pregnant or breastfeeding women were not included in this study owing to safety concerns. The metabolomic data were collected using cluster sampling from all the communities in one administrative subdistrict of Xi'hu District (*n* = 250). Details of the recruitment strategy and sampling scheme have been reported previously (20, 21). The study was approved by the Institutional Review Boards of Zhejiang University, Hangzhou, China (No. ZGL201507-3) and Stanford University, CA, USA (IRB-35020).

### Baseline Information Collection

At the baseline visit, we collected anthropometric data, including height, weight, and waist and hip circumference, and we conducted physical examinations and in-person interviews. The interviews addressed information on social-economic status, disease and medical history, physical activity, smoking and alcohol drinking habits, and a 26-item food frequency questionnaire for the dietary survey (22). The calculation of energy and nutrient intakes is reported elsewhere (23). Educational level was grouped into illiterate or primary school, middle or high school, or college or above. Physical activity was categorized as low, moderate, or high. Current smoking or alcohol drinking status was grouped into yes or no. For the biospecimen collection, we collected 12-h fasting blood samples on the same day as the baseline visit. The collected samples were processed within hours and stored immediately at −80°C for future lipid biomarker and metabolomic assessment. Dyslipidemia was determined according to the self-reported history of physician diagnosis or blood tests during baseline lipid biomarker examination. We defined dyslipidemia, according to the Guidelines for Prevention and Control of Dyslipidemia in Chinese Adults, as increased triglycerides (≥1.70 mmol/L), total cholesterol (≥5.18 mmol/L), low-density lipoprotein cholesterol (≥3.37 mmol/L), and decreased HDL cholesterol (<1.04 mmol/L) (24).

### Metabolomic Assessment

Untargeted metabolomic assays were carried out by Calibra Diagnostics/Metabolon using participant plasma samples and Metabolon's HD4 Discovery untargeted metabolomics platform. All samples were prepared using the automated MicroLab STAR system^®^ (Hamilton Company, Reno, NV, USA) and examined using ultra-performance liquid chromatography-tandem mass spectroscopy methods. Quality control standards were added to each sample extract to monitor the instrument performance and aid in a chromatographic alignment. Metabolites were identified by comparing them with the Metabolon library of purified standards or recurrent unknown entities based on retention time and index, mass-to-charge ratio, and chromatographic data. The plasma retinol level determination was added to the metabolomic assessment as the same method (ultra-performance liquid chromatography-tandem mass spectroscopy) was used for a regular determination (25). For plasma retinol levels, we used the standardized and centralized ratio of the area under the curve of retinol.

### Statistical Analysis

Continuous variables are presented as mean ± SD, and categorical variables are presented in numbers and percentages. There are sex differences in plasma retinol levels; therefore, the data were analyzed separately for men and women throughout this study. One male participant with a very high retinol level (higher than the upper quartile + the interquartile range) was treated as an outlier and excluded from the final analysis. The Shapiro–Wilk test was performed to check the normality and equality of variables. The variables that were not normally distributed were log-transformed to meet normality. If normality and equality did not meet after log transformation, the Kruskal–Wallis test was used for multiple comparisons and the value of *p* was adjusted for the false discovery rate (FDR) for a comparison between the groups.

Participants were divided into three groups according to the tertiles of plasma retinol level. ANOVA was used for multiple comparisons, and the Tukey test was used for a comparison between the groups. In the case of categorical variables, the chi-squared test was used for multiple comparisons, and FDR was used to adjust the value of *p* for comparisons between the groups. A logistic regression model was conducted to investigate the associations of plasma retinol level and dyslipidemia and was adjusted for age, educational level, physical activity, body mass index (BMI), current smoking, current alcohol drinking, dyslipidemia medication, vitamin A intake, and for women, menopausal status.

A linear regression model was used to investigate the association of age with the plasma retinol level. A multiple regression model was used to investigate an association between creatinine as a marker of kidney function, uric acid as an inflammatory biomarker, and plasma retinol, and was adjusted for age, educational level, physical activity, BMI, current smoking, current alcohol drinking, dyslipidemia medication, vitamin A intake, and menopausal status for women.

The ANCOVA was applied to the identification of metabolites related to plasma retinol groups and was adjusted for potential confounders, including age, educational level, physical activity, BMI, vitamin A intake, dyslipidemia medication, current smoking, current alcohol drinking, and menopausal status for women. FDR was used for the adjustment of values of *p* after conducting ANCOVA to avoid type I errors in metabolomic analyses. The *p* for trend was calculated among the three retinol tertiles for all the identified metabolites and was adjusted for FDR. All statistical analyses were completed using R version 3.6.2. To better understand the metabolomic profiles that are linked with the altered plasma retinol levels, the identified metabolites with a Human Metabolon Database (HMDB) ID were matched to metabolomic pathways using the pathway analysis features in MetaboAnalyst 4.0 (26). The global test method was used in a pathway enrichment analysis, and relative betweenness centrality was used to measure node importance in a topological analysis. Significance was set at *p* < 0.05 (two-tailed).

## Results

The baseline participants' characteristics are shown in Table 1. The average age of study participants was 50.5 years among men (*n* = 119) and 50.3 years among women (*n* = 130). There were no significant differences in terms of age, marital status, BMI, and vitamin A intake between men and women. Compared with women, men had higher educational levels, physical activity, and energy intake. Approximately 60.3% of male participants were current smokers, and no women smoked.

**Table 1:** Baseline characteristics of study participants.

	**Total** **(***n*** = 249)**	**Men** **(***n*** = 119)**	**Women** **(***n*** = 130)**	***p*** **-value**
Age (years), mean (SD)	50.4 (13.8)	50.5 (14.0)	50.3 (13.6)	0.910
Marital status, % (*n*)				0.341
Single	4.1 (10)	4.3 (5)	3.9(5)	
Married	93.0 (226)	93.1 (108)	92.9 (118)	
Divorced	0.8 (2)	1.7 (2)	0.00 (0)	
Widowed	2.1 (5)	0.9 (1)	3.1 (4)	
Educational level, % (*n*)				<0.001
Illiterate or primary school	46.5 (113)	32.8 (38)	59.1 (75)	
Middle school or high school	39.5 (96)	52.6 (61)	27.6 (35)	
College or above	14.0 (34)	14.7 (17)	13.4 (17)	
Physical activity, %(*n*)				0.023
Low	21.1 (51)	21.7 (25)	20.5 (26)	
Moderate	55.8 (135)	47.8 (55)	63.0 (80)	
High	23.18 (56)	30.4 (35)	16.5 (21)	
Current smoke, %(*n*)	28.88 (70)	60.3 (70)	0.0 (0)	<0.001
Current alcohol drinking, % (*n*)	48.7 (118)	69.6 (80)	29.9 (38)	<0.001
BMI	23.5 (3.17)	23.9 (3.31)	23.2 (3.00)	0.063
Dyslipidemia, % (*n*)	58.4 (142)	64.7 (75)	52.8 (67)	0.080
**Related energy or nutrients intake (1st quantile, 3rd quantile)**				
Total energy, kcal	1,430.7 (1,098.2, 1,837.4)	1,664.4 (1,286.9, 2,112.5)	1,286.0 (981.2, 1,557.2)	<0.001
Vitamin A intake, μg RE	510.5 (307.7, 784.5)	503.2 (280.3, 775.1)	510.5 (319.3, 789.7)	0.547

*Results are presented in % (n) for categorical variables, mean ± SD for parametric continuous variables, and median (1st quartile and 3rd quartile) for non-parametric continuous variables*.

*The values of p were calculated using ANOVA for parametric continuous variables, the Kruskal–Wallis-test for non-parametric continuous variables, and the chi-squared test or Fisher's exact-test for categorical variables*.

*SD, standard deviation; BMI, body mass index; RE, retinol equivalent*.

An overview of the baseline information in different retinol tertile groups by sex is shown in Supplementary Table 1. For men, no significant differences were observed in terms of age, marital status, educational level, physical activity, current smoking, BMI, total energy intake, and dietary vitamin A intake among the three plasma retinol groups, whereas a significantly higher percentage of participants with current alcohol drinking was found in the 3rd retinol tertile group in comparison with the 1st tertile. For women, no significant difference was found in marital status, physical activity, BMI, total energy intake, and dietary vitamin A intake among the three plasma retinol groups; however, the 3rd tertile group had a significantly higher age and lower educational level.

**Figure 1:**
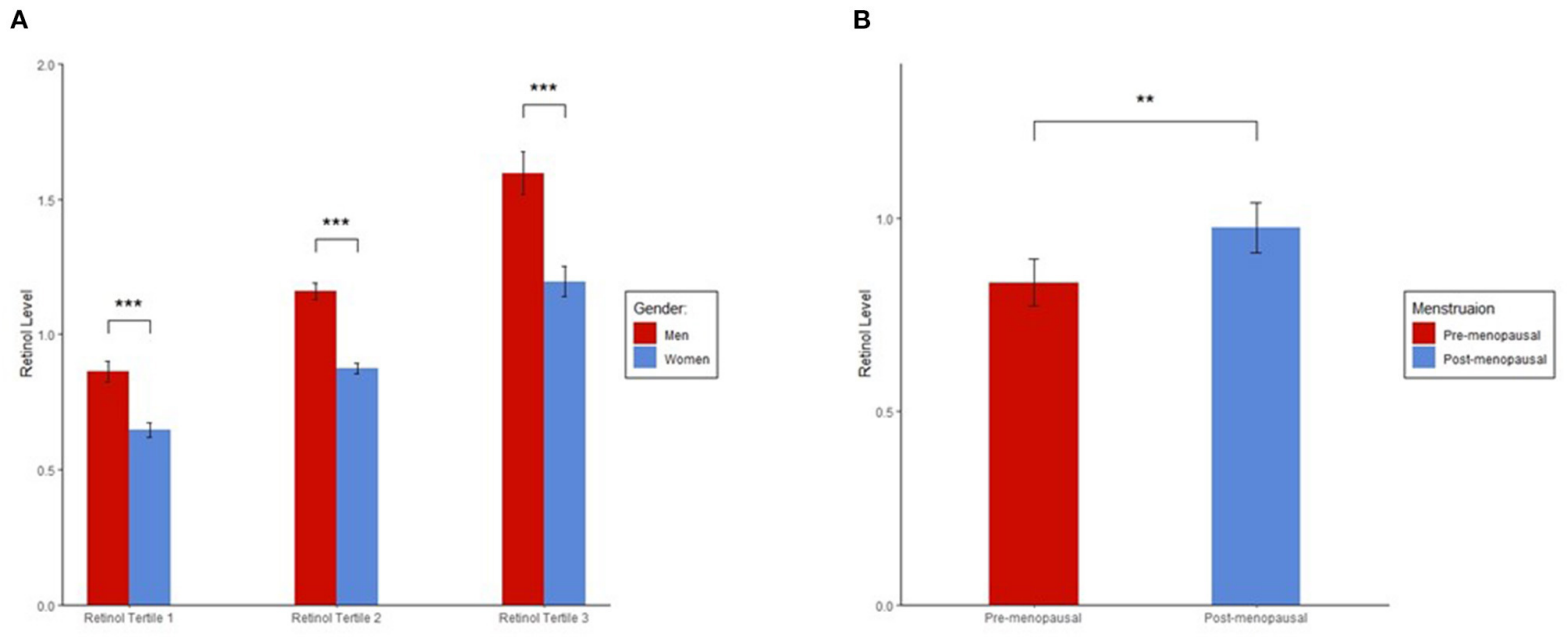
**(A)** Sex differences in plasma retinol levels. **(B)** Plasma retinol levels in pre and post-menopausal women. Plasma retinol levels in each group were presented as mean ± 95% CI. ANOVA was used to determine significant differences in retinol levels between the groups. Plasma retinol levels were log-transformed to a normal distribution in ANOVA. We found significant differences in plasma retinol levels for each tertile between men and women and in plasma retinol levels between pre and post-menopausal women. ^***^p < 0.001; ^**^p < 0.01.

We found a significant difference in plasma retinol levels between men and women (Figure 1A). In each tertile group, men had significantly higher retinol levels than women. Among women, ~48.8% were post-menopausal, and a significantly higher plasma retinol level was found in post-menopausal than pre-menopausal women (*p* = 0.001; Figure 1B).

**Figure 2:**
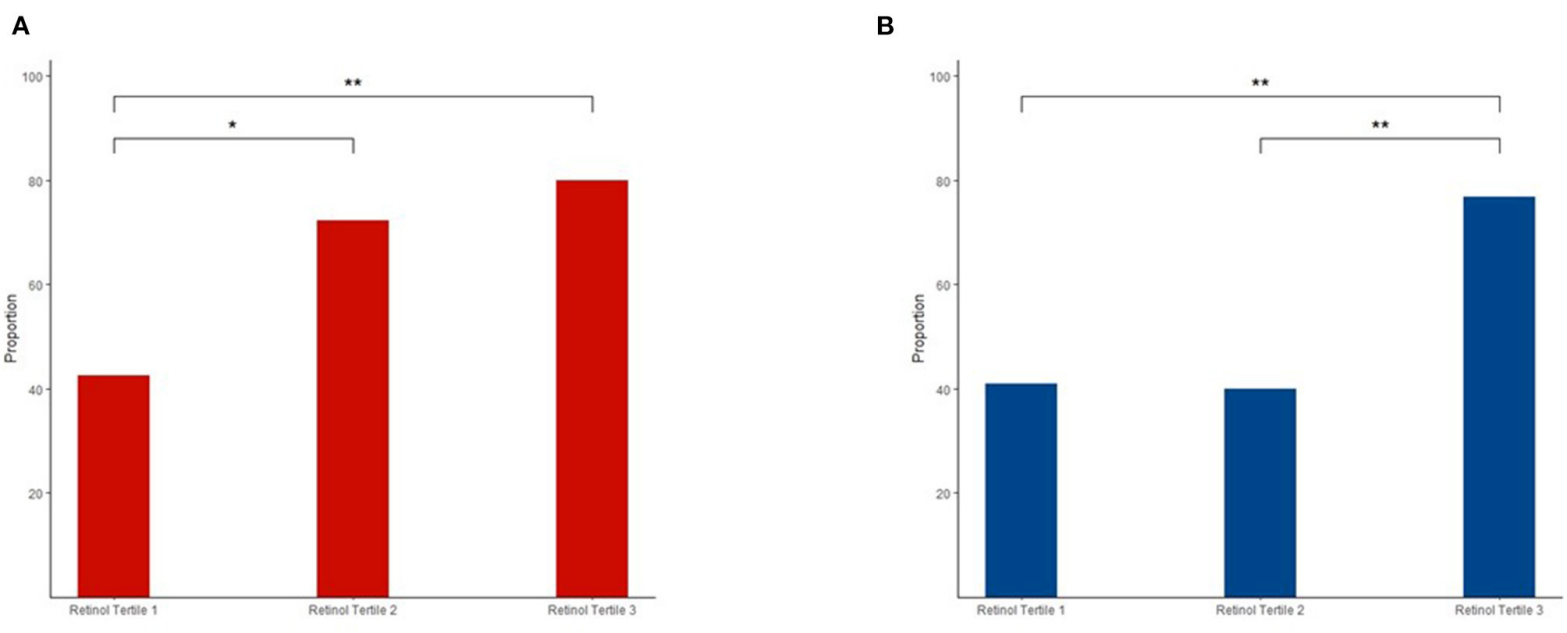
Different proportions of dyslipidemia in the three retinol tertiles among men **(A)** and women **(B)**. The chi-squared-test was used for comparisons, and the false discovery rate (FDR) was used to adjust the value of *p* for comparisons between the groups. ^**^p < 0.01; ^*^p < 0.05.

Figure 2 shows the significant associations between plasma retinol tertiles and dyslipidemia. In men, the 2nd and 3rd tertiles showed significantly higher proportions of dyslipidemia than the 1st tertile (1st tertile vs. 2nd tertile: *p* = 0.026; 1st tertile vs. 3rd tertile: *p* = 0.003). In women, the 3rd tertile had significantly higher proportions of dyslipidemia than the 1st and 2nd tertiles (3rd tertile vs. 1st tertile: *p* = 0.002, 3rd tertile vs. 2nd tertile: *p* = 0.002). We conducted a logistic regression model adjusted for potential confounders, and the results were consistent (Supplementary Table 2). Significant positive associations among age, creatinine, uric acid, and plasma retinol levels were only found in women (age: β = 0.007, *p* < 0.001; creatinine: β = 0.1, *p* = 0.009; and uric acid: β = 0.3, *p* = 0.002) but not in men (age: β = 0.003, *p* = 0.137; creatinine: β = 0.02, *p* = 0.761; and uric acid: β = 0.1, *p* = 0.123).

In the case of untargeted metabolomic assessment, 751 metabolites were detected. A total of 75 and 30 metabolites between a comparison of the 1st and 3rd retinol tertiles were identified in men and women, respectively. No significantly different metabolites were found between the 1st and 2nd retinol tertiles and between the 2nd and 3rd retinol tertiles in both men and women. Among the abovementioned 75 and 30 metabolites, 10 identical metabolites were identified in both men and women, all of them were from the lipid super pathway group (Table 2). The most abundant metabolites were from the phosphatidylcholine (PC) sub-pathway group, followed by androgenic steroids, lysophospholipid, and phosphatidylethanolamine subpathway groups.

**Table 2:** Metabolites were identified using ANCOVA between 1st and 3rd retinol tertiles in both men and women.

**Biochemical**	**Super pathway**	**Sub-pathway group**
Androstenediol (3beta, 17beta) disulfate	Lipid	Androgenic steroids
Androstenediol (3beta, 17beta) monosulfate	Lipid	Androgenic steroids
Androstenediol (3beta, 17beta) monosulfate	Lipid	Androgenic steroids
1-Palmitoyl-GPC (16:0)	Lipid	Lysophospholipid
1-Stearoyl-GPE (18:0)	Lipid	Lysophospholipid
1-Stearoyl-2-arachidonoyl-GPC (18:0/20:4)	Lipid	Phosphatidylcholine (PC)
1-palmitoyl-2-arachidonoyl-GPC (16:0/20:4n6)	Lipid	Phosphatidylcholine (PC)
1-Linoleoyl-2-arachidonoyl-GPC (18:2/20:4n6)	Lipid	Phosphatidylcholine (PC)
1-myristoyl-2-arachidonoyl-GPC (14:0/20:4)	Lipid	Phosphatidylcholine (PC)
1-Stearoyl-2-arachidonoyl-GPE (18:0/20:4)	Lipid	Phosphatidylethanolamine (PE)

*GPC, glycerophosphorylcholine; GPE, glycerophosphatidylethanolamine*.

All the identified metabolites in men (75 metabolites) and women (30 metabolites) are presented in Supplementary Tables 3, 4, respectively. All the detected metabolites in men and women had a *p* for trend <0.05. In both men and women, the lipid super pathway was the most involved. In men, 62 metabolites were identified, with the leading subpathway groups being lysophospholipid, PC, phosphatidylethanolamine, polyunsaturated fatty acids (n-3 and n-6), sphingomyelins, and androgenic steroids. Other metabolites were only observed in the amino acid and xenobiotic super pathway groups. Seven metabolites belonged to the xenobiotic super pathway group, in which the four metabolites were in the xanthine metabolism subpathway group. The amino acid super pathway group included six metabolites belonging to the leucine, isoleucine, and valine metabolism, glutathione metabolism, urea cycle, arginine, and proline metabolism subpathway groups. In women, although fewer metabolites were identified, super and subpathway groups of these metabolites were no less complex than those identified in men. From the lipid super pathway group, 18 metabolites were included. The most abundant metabolites were in a subpathway group of androgenic steroids, followed by PC and pregnenolone steroids. The metabolites in the amino acid super pathway group were more complex in women than in men. Metabolites were identified in the tryptophan metabolism, leucine, isoleucine, and valine metabolism, creatinine metabolism, urea cycle, arginine, and proline metabolism subpathway groups. Only one metabolite was identified in the xenobiotic super pathway group among women. We found metabolites as cofactors and vitamins and in the nucleotide, and peptide super pathway groups, but these super pathway groups were not observed in men. Nearly, all the metabolites were upregulated in both men and women, except the case of two [dehydroisoandrosterone sulfate (DHEA-S) and pregnenediol sulfate (C_21_H_34_O_5_S)] that were downregulated in women.

**Figure 3:**
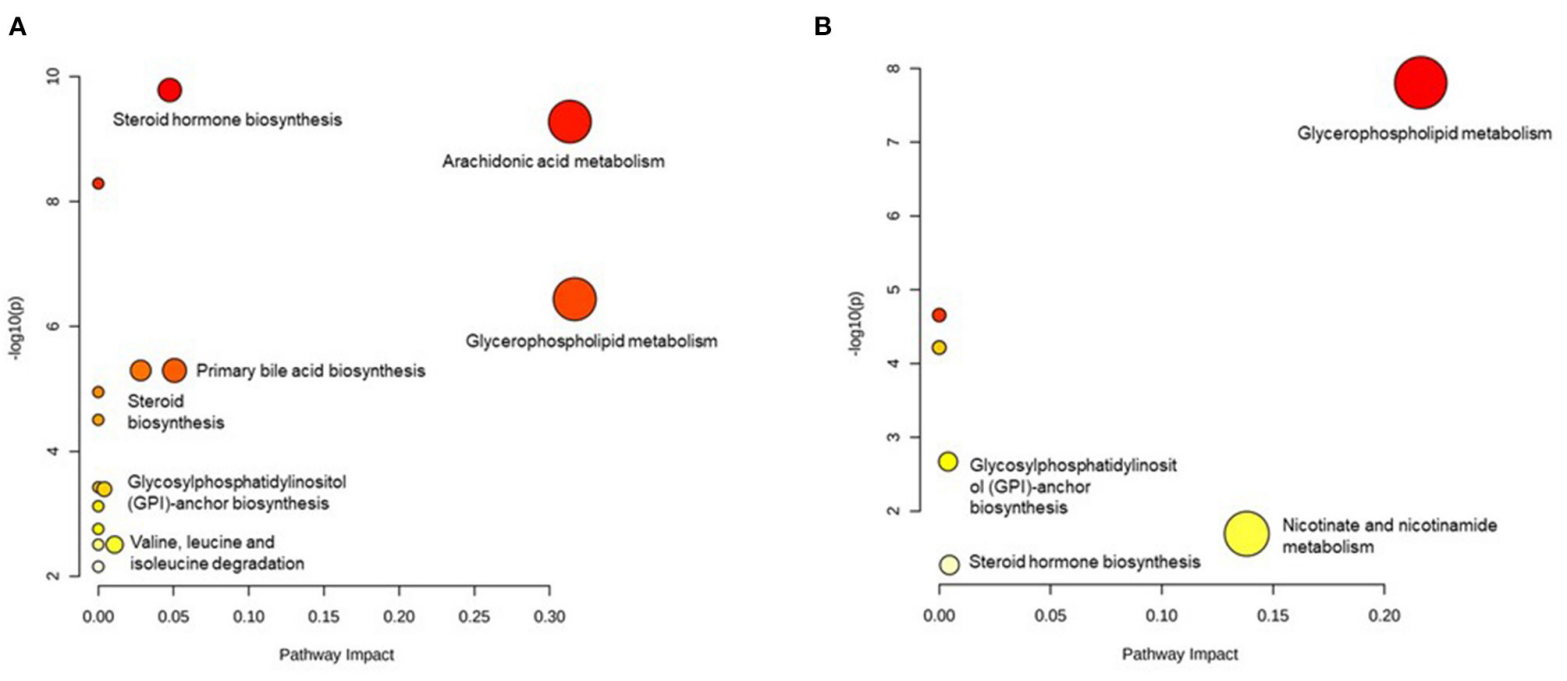
Pathways identified in men **(A)** and women **(B)** using pathway analysis.

We excluded those metabolites that are without an HMDB ID; therefore, 64 metabolites in men and 22 metabolites in women were included in the pathway analysis. In men, 16 pathways were detected and the pathways with a high impact were arachidonic acid metabolism, glycerophospholipid metabolism, primary bile acid biosynthesis, steroid hormone biosynthesis, steroid biosynthesis, valine, leucine, isoleucine degradation, and glycosylphosphatidylinositol- (GPI-) anchor biosynthesis (Figure 3A). In women, eight pathways were identified. The pathways with a high impact were glycerophospholipid metabolism, nicotinate, and nicotinamide metabolism, steroid hormone biosynthesis, and GPI-anchor biosynthesis (Figure 3B). From all the identified pathways (Supplementary Tables 5, 6), those that overlapped across the sexes were glycerophospholipid metabolism, arachidonic acid metabolism, linoleic acid metabolism, alpha-linolenic acid metabolism, steroid hormone biosynthesis, and GPI-anchor biosynthesis.

## Discussion

To the best of our knowledge, this was the first study to investigate the associations among plasma retinol, dyslipidemia, and related metabolomic profiles. In this study, although plasma retinol levels showed sex differences, we found that a higher level of plasma retinol increased the risk of dyslipidemia in both sexes. We also examined the associated metabolomic profiles and found that the same metabolites and pathways were changed in both men and women, and most of them were related to lipid metabolism. The overlapped pathways showed hormone-regulated, inflammatory, and oxidative stress-derived pathways, and sex-specific pathways were also found. This study provides insights into the complex role of plasma retinol in the lipid profile.

Despite the sex differences in the plasma retinol level, similar associations between plasma retinol and dyslipidemia were observed in both men and women. Some differences between plasma retinol and dyslipidemia also existed, which were confirmed by using the metabolomic data. In men, higher proportions of dyslipidemia were noted in the 2nd and 3rd retinol tertiles whereas, in women, this trend was found in the 3rd tertile. Logistic regression also showed increased odds of dyslipidemia in the 2nd and 3rd plasma retinol tertiles in men, and increased odds in the 3rd plasma retinol tertile compared with the 1st and 2nd tertiles in women. This finding implies that when plasma retinol is increased to a certain level, an association with a higher risk of dyslipidemia exists regardless of sex. Serum or plasma retinol reflects vitamin A nutritional status in humans, which is precisely maintained within a narrow range (27). It has been shown that women have lower mean plasma retinol than men, indicating lower retinol storage in women or different retinol metabolism according to sex (28, 29). We found positive associations among creatinine, uric acid, and plasma retinol in women but not in men, which also indicated differences in metabolic change by sex. In our study, we also found that most of the identified metabolites related to steroid hormones belonged to the androgen steroid hormone subpathway group and the pathway of steroid hormone biosynthesis in both men and women, which implies that the hormone might affect the metabolism of retinol.

Despite the sex difference in plasma retinol, steroid hormone biosynthesis itself is also an important pathway that interacts with both plasma retinol and dyslipidemia. In both animal and cell studies, the upregulation of retinoids would cause the regulation of steroid hormone biosynthesis and, thereby maintain the steroid level required for physiological activities that diminish with aging (30). This might be because of the mediation of retinoid signaling nuclear receptors, retinoid X receptors, and retinoic acid receptors, which are also in the steroid/thyroid hormone receptor superfamily and are expressed in varying amounts in steroidogenic tissues (31). Several human studies have focused on sex hormones and serum retinol in women, but to our knowledge, little such research has been done in men. Most studies have focused on estradiol in women, but the results have been inconsistent (32, 33). Only one study focusing on patients with polycystic ovary syndrome showed that retinol-binding protein 4, which was considered to be highly correlated with serum retinol (34), was positively related to androgen hormones (35). Generally, it is well-understood that androgens have a negative influence on the lipid profile in men, and estrogens have a positive influence in women (36). We also observed the downregulation of estrogen-related metabolites in women, as well as significantly higher retinol levels in post-menopausal women and a positive association of age with plasma retinol. All this information indicated that the upregulation of plasma retinol in participants with dyslipidemia may be significantly associated with hormone synthesis.

The only identified two downregulated metabolites, DHEA-S and pregnenediol sulfate (C_21_H_34_O_5_S), in women were both related to steroid hormone metabolism. Although studies on an association between plasma retinol and pregnenediol sulfate (C_21_H_34_O_5_S) are lacking, DHEA or DHEA-S, one of the most abundant circulating steroids in humans, is a precursor for estrogen and androgen production and naturally declines with age. Low plasma DHEA-S has been strongly associated with dyslipidemia and increases the risk for CVD (37). The plasma retinol-associated DHEA downregulation found in our study might be associated with an increased risk of dyslipidemia in women.

Women had only one pathway, nicotinate and nicotinamide metabolism, which was not identified in men. The pathways in men were much more complicated than those in women, with primary bile acid biosynthesis, valine, leucine, and isoleucine degradation as higher impact pathways. Nicotinate has been used to treat dyslipidemia since 1950 because it can reduce triglycerides and low-density lipoprotein cholesterol and raise HDL cholesterol (38). Primary bile acids are the ligands or activators of some promiscuous receptors integrating lipid homeostasis with xenobiotic metabolism, and they exert synergistic activities in regulating lipid and glucose homeostasis (39). Valine, leucine, and isoleucine all are branched-chain amino acids (BCAAs). An increase in plasma BCAAs is considered a potential biomarker of metabolic diseases, such as insulin resistance, type 2 diabetes mellitus, and CVD (40). A study in Japan also found that plasma BCAAs were positively associated with dyslipidemia (41). The degradation of BCAAs might have a reverse effect. All these pathways seem to have a protective effect against dyslipidemia, which suggests that an increase in retinol might be caused by dyslipidemia. However, as we did not identify these pathways in both the sexes, further study with larger sample size is needed to clarify the associations.

In our study, we found metabolites and metabolic pathways that overlapped between men and women and that could be used to distinguish low from high retinol levels, apart from steroid hormone biosynthesis. Among the overlapped pathways, the most influential pathway was glycerophospholipid metabolism, followed by arachidonic acid metabolism, linoleic acid metabolism, alpha-linolenic acid metabolism, and GPI-anchor biosynthesis. Glycerophospholipid metabolism identified in our study was also associated with the four types of CVD and may, therefore, be a key point in CVD (42). Consistent with our study, a study focusing on patients with dyslipidemia found that glycerophospholipid metabolism was reported to be the most significantly enriched pathway in patients (19). However, in both of these studies, glycerophospholipid-related metabolites, such as PC, phosphatidylethanolamine, and lysophospholipid, were downregulated; compared with our results, these metabolites were upregulated in a higher retinol group. It has been reported that the downregulation of PC might promote macrophage activation *via* cell signaling, leading to chronic inflammatory responses (43–45). Retinol is considered an indirect antioxidant that influences gene expression to increase effective antioxidant responses (46); thus, our study results suggest that increased plasma retinol interacts with an upregulated PC owing to the needs of body tissues in a dyslipidemic condition.

Other metabolites, such as arachidonic acid metabolism and linoleic acid metabolism pathways, found in both the sexes also suggested that increased inflammation might occur in the highest retinol group. Arachidonic acid and linoleic acid both are *n*-6 polyunsaturated fatty acids, which are essential fatty acids for humans. However, arachidonic acid and the precursor linoleic acid, is a precursor of pro-inflammatory factors (47). The metabolites involved in these two pathways might be highly bioactive and contribute to the establishment of oxidative stress and unresolved chronic inflammation, and may eventually be involved in the emergence and course of cardiovascular and metabolic diseases (48, 49). A previous study also reported a complicated association between inflammatory biomarkers and serum retinol in patients with CVD (50). That study suggested a potential interplay between inflammatory processes, serum retinol, and atherosclerosis, which was also found among inflammation, plasma retinol, and dyslipidemia in our study. It is plausible that an association between dyslipidemia and an increase in plasma retinol observed in our study might be caused by the compensation of plasma retinol under the conditions that arise from inflammation and oxidative stress because plasma retinol is considered as an antioxidant.

Elevated plasma retinol is involved in the mobilization of retinol storage and related metabolic changes associated with dyslipidemia, which might be a key point in such an increase. We did not observe any differences in dietary vitamin A intake among the three plasma retinol tertile groups. In an animal study, a dietary retinoid intake was the only nutritional factor to affect retinol storage (51); thus, the change in the mobilization of retinol storage affected retinol in a bloodstream. In the human body, 80% of retinol is contained in lipid droplets reserved in hepatic stellate cells in the form of retinyl esters (52). Older patients with ischemic CVD have inadequate liver storage of retinol (53). One study also suggested that this might be caused by the mobilization of hepatic storage of vitamin A for target tissues that might need more antioxidants to deal with oxidative damage owing to an altered lipid profile (17). Future intervention studies may need to focus on a diet that is high in vitamin A or a vitamin A supplementation in patients with dyslipidemia and high serum retinol levels, especially in men, to yield a deeper understanding of the role of vitamin A in the lipid profile.

This study has several merits and limitations. First, it was the first population-based study to focus on plasma retinol and dyslipidemia in the general population. Second, our study used high-resolution untargeted metabolomics assays to precisely investigate metabolomic profiles and pathways associated with the plasma retinol level. Moreover, we considered sex differences in analyzing the association of plasma retinol with dyslipidemia and the identification of related metabolites and pathways. Both overlapping and sex-specific metabolites and pathways were identified in our study. As for the limitations of this study, first, we only used the ratio of the area under the curve rather than the exact retinol concentration. Therefore, the exact concentrations of retinol could not be determined. However, the ratio of the area under the curve can reflect the concentration in ultra-performance liquid chromatography-tandem mass spectroscopy when detecting metabolites, including retinol (54). Additionally, we did not include dietary supplement surveys in the cohort baseline; therefore, diet vitamin A intake might be underestimated in some participants. Second, the sample size in this study was small, and some potential confounders such as alcohol drinking status could not be fully adjusted. Further studies with larger and different populations are needed. Additionally, this was a cross-sectional study; future longitudinal studies are needed to determine causality.

In conclusion, our findings suggested that, despite a difference in plasma retinol levels between men and women, a positive association between plasma retinol and dyslipidemia exists in both sexes. The results of metabolomic profile and pathway analyses showed that steroid hormone biosynthesis might play an important role. Other pathways, such as glycerophospholipid metabolism, showed that an association between plasma retinol and dyslipidemia might be inflammatory and related to oxidative stress, and increased plasma retinol may be associated with compensation. In addition, large, population-based longitudinal studies and intervention studies are needed to confirm plasma retinol as an important factor in dyslipidemia and CVD prevention.

## Data Availability Statement

The original contributions presented in the study are included in the article/Supplementary Material, further inquiries can be directed to the corresponding authors.

## Ethics Statement

The studies involving human participants were reviewed and approved by the Institutional Review Boards of the Zhejiang University, Hangzhou, China (No. ZGL201507-3) and the Stanford University, CA, USA (IRB-35020). The patients/participants provided their written informed consent to participate in this study.

## Author Contributions

NW, YR, AH, and SZ designed this study, responsible for the methodology, and the WELL China team carried out a field work investigation. NW analyzed the data. NW, YR, and ZY wrote the manuscript and created the tables and figures. SZ and AH supervised all processes of the projects and provided constructive suggestions. NW, YR, ZY, CS, SL, YM, XZ, YL, AH, and SZ contributed to the drafting and critical revision of the manuscript for intellectual content. All authors read and revised the manuscript and approved the final submitted version.

## Funding

This study was supported by the Nutrilite Health Institute Wellness Fund, Cyrus Tang Foundation, Zhejiang University Education Foundation, and the Hsun K. Chou Fund of Zhejiang University Education Foundation.

## Conflict of Interest

The authors declare that the research was conducted in the absence of any commercial or financial relationships that could be construed as a potential conflict of interest.

## Publisher's Note

All claims expressed in this article are solely those of the authors and do not necessarily represent those of their affiliated organizations, or those of the publisher, the editors and the reviewers. Any product that may be evaluated in this article, or claim that may be made by its manufacturer, is not guaranteed or endorsed by the publisher.

## Abbreviations

CDV: cardiovascular disease
HDL: high-density lipoprotein
FDR: false discovery rate
BMI: body mass index
HMDB: Human Metabolon Database
PC: phosphatidylcholine
DHEA-S: dehydroisoandrosterone sulfate
GPI: glycosylphosphatidylinositol
BCAAs: branched-chain amino acids.
