# Supplementary material for: Metabolomic Profiles of Plasma Retinol-Associated Dyslipidemia in Men and Women
Source: Front Nutr. 2021 Nov 17;8:740435. doi: 10.3389/fnut.2021.740435 (PMC8635783; doi:10.3389/fnut.2021.740435)
Supplement: Supplementary file 1 [file Table_1.DOCX]

**Table S1 Baseline characteristics of retinol tertiles in both men and women.**

|  | Men | | | | Women | | | |
| --- | --- | --- | --- | --- | --- | --- | --- | --- |
|  | Retinol tertile 1 (n=40) | Retinol tertile 2 (n=39) | Retinol tertile 3 (n=40) | p-value | Retinol tertile 1 (n=44) | Retinol tertile 2 (n=43) | Retinol tertile 3 (n=43) | p-value |
| Age (years), mean (SD) | 49.8 (15.8) | 47.4 (12.8) | 54.2 (12.4) | 0.088 | 44.5 (12.3) | 48.7 (13.7)^b^ | 60.00 (11.45)^c^ | **<0.001** |
| Marital status, %(n) |  |  |  | 0.429 |  |  |  | 0.368 |
| Single | 7.5 (3) | 5.4 (2) | 0.0 (0) |  | 2.3 (1) | 7.3 (3) | 2.38 (1) |  |
| Married | 90.0 (36) | 94.6 (35) | 94.9 (37) |  | 97.7 (43) | 87.8 (3) | 92.86 (39) |  |
| Divorced | 2.5 (1) | 0.0 (0) | 2.6 (1) |  | 0.00 (0) | 0.0 (0) | 0.00 (0) |  |
| Widowed | 0.0 (0) | 0.0 (0) | 2.6 (1) |  | 0.00 (0) | 4.9 (2) | 4.76 (2) |  |
| Educational level, %(n) |  |  |  | 0.602 |  |  |  | **0.001** |
| Illiterate or primary school | 30.0 (12) | 35.1 (13) | 33.3 (13) |  | 36.4 (16) | 61.0 (25) | 80.95 (34) |  |
| Middle school or high school | 52.5 (21) | 46.0 (17) | 59.0 (23) |  | 47.7 (21) | 22.0 (9) | 11.90 (5) |  |
| College or above | 17.5 (7) | 18.9 (7) | 7.7 (3) |  | 15.9 (7) | 17.0 (7) | 7.14 (3) |  |
| Physical activity, %(n) |  |  |  | 0.996 |  |  |  | 0.126 |
| Low | 22.5 (9) | 19.4 (7) | 23.1 (9) |  | 27.3 (12) | 14.6 (6) | 19.05 (8) |  |
| Moderate | 47.5 (19) | 50.0 (18) | 46.2 (18) |  | 52.3 (23) | 78.1 (32) | 59.52 (25) |  |
| High | 30.0 (12) | 30.6 (11) | 30.8 (12) |  | 20.5 (9) | 7.3 (3) | 21.43 (9) |  |
| Current smoke, %(n) | 65.0 (26) | 54.1 (20) | 61.5 (24) | 0.607 | 0.00 (0) | 0.0 (0) | 0.00 (0) | - |
| Current alcohol drinking, %(n) | 57.5 (23) | 61.1 (22) | 89.7 (35)^c^ | **0.003** | 27.3 (12) | 31.7 (13) | 31.0 (13) | 0.891 |
| BMI | 24.2 (3.8) | 23.3 (3.4) | 24.3 (2.6) | 0.354 | 22.3 (3.2) | 23.5 (3.1) | 23.79 (2.5) | 0.053 |
| Dyslipidemia, %(n) | 42.5 (17)^a^ | 72.2 (26) | 80.0 (32)^c^ | **0.001** | 40.9 (18) | 40.0 (16)^b^ | 76.7 (33)^c^ | **<0.001** |
| *Related energy or nutrients intake (1^st^ quantile, 3^rd^ quantile)* |  |  |  |  |  |  |  |  |
| Total energy intake, kcal | 1636.3 (1261.2, 2181.3) | 1659.5 (1241.4, 2008.9) | 1741.1 (1453.4, 2417.6) | 0.555 | 1399.5 (1092.2, 1667.1) | 1210.4 (971.2, 1420.5) | 1272.7 (945.9, 1537.2) | 0.210 |
| Vitamin A intake, μg RE | 523.7 (327.7, 897.3) | 442.0 (273.1, 840.5) | 500.1 (284.8, 692.3) | 0.731 | 427.9 (311.1, 899.7) | 487.8 (363.3, 696.1) | 593.5 (294.7, 803.2) | 0.968 |

1. Results are presented as % (n) for categorical variables, mean±SD for parametric continuous variables, and median (first quartile, third quartile) for non-parametric continuous variables.
2. P-values were calculated using ANOVA for parametric continuous variables, Kruskal–Wallis test for non-parametric continuous variables, and chi-square test or Fisher’s exact test for categorical variables. For comparisons between groups, the false discovery rate was used for adjustment.
3. a: There was significant difference between retinol tertile 1 and retinol tertile 2 (p < 0.05).

b: There was significant difference between retinol tertile 2 and retinol tertile 3 (p < 0.05).

c: There was significant difference between retinol tertile 1 and retinol tertile 3 (p < 0.05).

1. SD, standard deviation; BMI, body mass index; RE, retinol equivalent.

**Table S2** **Logistic regression model for dyslipidemia and plasma retinol tertiles by sex.**

|  | Men | | | Women | | |
| --- | --- | --- | --- | --- | --- | --- |
|  | OR | 95% CI | p-value | OR | 95% CI | p-value |
| 2^nd^ Vs. 1^st^ tertile | 13.8 | 3.3, 57.3 | **< 0.001** | 0.9 | 0.2, 3.6 | 0.824 |
| 3^rd^ Vs. 1^st^ tertile | 12.6 | 3.2, 49.8 | **< 0.001** | 7.2 | 1.3, 39.8 | **0.023** |
| 3^rd^ Vs. 2^nd^ tertile | 0.9 | 0.2, 3.8 | 0.899 | 8.5 | 2.1, 35.0 | **0.003** |

1. The model was adjusted for age, educational level, physical activity, body mass index, dyslipidemia medication, vitamin A intake, alcohol drinking, current smoking, and menopause status (women).
2. OR, odds ratio; CI, confidence interval.

**Table S3 Significant metabolites in 1st and 3rd retinol tertiles among men.**

| No. | Biochemical | Super pathway | Sub pathway group | HMDB ID | β | P-adjusted |
| --- | --- | --- | --- | --- | --- | --- |
| 1 | 2-hydroxybutyrate/2-hydroxyisobutyrate | Amino Acid | Glutathione Metabolism | NA | 0.29 | 0.033 |
| 2 | cys-gly, oxidized | Amino Acid | Glutathione Metabolism | NA | 0.27 | 0.043 |
| 3 | 4-methyl-2-oxopentanoate | Amino Acid | Leucine, Isoleucine and Valine Metabolism | HMDB00695 | 0.15 | 0.034 |
| 4 | 3-hydroxy-2-ethylpropionate | Amino Acid | Leucine, Isoleucine and Valine Metabolism | HMDB00396 | 0.33 | 0.002 |
| 5 | 3-methylglutaconate | Amino Acid | Leucine, Isoleucine and Valine Metabolism | HMDB00522 | 0.24 | 0.048 |
| 6 | N2,N5-diacetylornithine | Amino Acid | Urea cycle; Arginine and Proline Metabolism | NA | 0.57 | 0.006 |
| 7 | 5alpha-androstan-3beta,17beta-diol disulfate | Lipid | Androgenic Steroids | HMDB00493 | 0.59 | 0.004 |
| 8 | androstenediol (3beta,17beta) disulfate (1) | Lipid | Androgenic Steroids | HMDB03818 | 0.81 | <0.001 |
| 9 | androstenediol (3beta,17beta) monosulfate (1) | Lipid | Androgenic Steroids | HMDB03818 | 0.39 | 0.011 |
| 10 | androstenediol (3beta,17beta) monosulfate (2) | Lipid | Androgenic Steroids | NA | 0.73 | 0.001 |
| 11 | cortisone | Lipid | Corticosteroids | HMDB02802 | 0.26 | 0.043 |
| 12 | cortisol | Lipid | Corticosteroids | HMDB00063 | 0.44 | 0.002 |
| 13 | palmitoylcarnitine (C16) | Lipid | Fatty Acid Metabolism(Acyl Carnitine) | HMDB00222 | 0.16 | 0.044 |
| 14 | stearoylcarnitine (C18) | Lipid | Fatty Acid Metabolism(Acyl Carnitine) | HMDB00848 | 0.28 | 0.002 |
| 15 | arachidonoylcarnitine (C20:4) | Lipid | Fatty Acid Metabolism(Acyl Carnitine) | NA | 0.29 | 0.045 |
| 16 | (16 or 17)-methylstearate (a19:0 or i19:0) | Lipid | Fatty Acid, Branched | HMDB37397 | 0.33 | 0.028 |
| 17 | 1-stearoyl-GPI (18:0) | Lipid | Lysophospholipid | HMDB61696 | 0.22 | 0.006 |
| 18 | 1-palmitoyl-GPC (16:0) | Lipid | Lysophospholipid | HMDB10382 | 0.26 | <0.001 |
| 19 | 1-stearoyl-GPC (18:0) | Lipid | Lysophospholipid | HMDB10384 | 0.26 | <0.001 |
| 20 | 1-oleoyl-GPC (18:1) | Lipid | Lysophospholipid | HMDB02815 | 0.32 | 0.002 |
| 21 | 1-stearoyl-GPE (18:0) | Lipid | Lysophospholipid | HMDB11130 | 0.29 | 0.001 |
| 22 | 1-palmitoleoyl-GPC (16:1)* | Lipid | Lysophospholipid | HMDB10383 | 0.39 | 0.001 |
| 23 | 1-arachidonoyl-GPC (20:4n6)* | Lipid | Lysophospholipid | HMDB10395 | 0.48 | <0.001 |
| 24 | 2-palmitoyl-GPC (16:0)* | Lipid | Lysophospholipid | HMDB61702 | 0.20 | 0.014 |
| 25 | 1-arachidonoyl-GPE (20:4n6)* | Lipid | Lysophospholipid | HMDB11517 | 0.29 | 0.001 |
| 26 | 1-arachidonoyl-GPI (20:4)* | Lipid | Lysophospholipid | HMDB61690 | 0.32 | 0.002 |
| 27 | 1-linolenoyl-GPC (18:3)* | Lipid | Lysophospholipid | HMDB10388 | 0.40 | 0.009 |
| 28 | 1-(1-enyl-palmitoyl)-GPE (P-16:0)* | Lipid | Lysoplasmalogen | NA | 0.30 | 0.003 |
| 29 | 1-(1-enyl-stearoyl)-GPE (P-18:0)* | Lipid | Lysoplasmalogen | NA | 0.33 | 0.002 |
| 30 | 1-(1-enyl-oleoyl)-GPE (P-18:1)* | Lipid | Lysoplasmalogen | NA | 0.41 | 0.001 |
| 31 | 1-stearoyl-2-arachidonoyl-GPC (18:0/20:4) | Lipid | Phosphatidylcholine (PC) | HMDB08048 | 0.37 | <0.001 |
| 32 | 1-stearoyl-2-oleoyl-GPC (18:0/18:1) | Lipid | Phosphatidylcholine (PC) | HMDB08038 | 0.36 | <0.001 |
| 33 | 1-palmitoyl-2-arachidonoyl-GPC (16:0/20:4n6) | Lipid | Phosphatidylcholine (PC) | HMDB07982 | 0.38 | <0.001 |
| 34 | 1-palmitoyl-2-docosahexaenoyl-GPC (16:0/22:6) | Lipid | Phosphatidylcholine (PC) | HMDB07991 | 0.27 | 0.003 |
| 35 | 1-palmitoyl-2-stearoyl-GPC (16:0/18:0) | Lipid | Phosphatidylcholine (PC) | HMDB07970 | 0.28 | 0.001 |
| 36 | 1-stearoyl-2-linoleoyl-GPC (18:0/18:2)* | Lipid | Phosphatidylcholine (PC) | HMDB08039 | 0.12 | 0.021 |
| 37 | 1-palmitoyl-2-palmitoleoyl-GPC (16:0/16:1)* | Lipid | Phosphatidylcholine (PC) | HMDB07969 | 0.43 | 0.013 |
| 38 | 1-oleoyl-2-docosahexaenoyl-GPC (18:1/22:6)* | Lipid | Phosphatidylcholine (PC) | HMDB08123 | 0.24 | 0.036 |
| 39 | 1-linoleoyl-2-arachidonoyl-GPC (18:2/20:4n6)* | Lipid | Phosphatidylcholine (PC) | HMDB08147 | 0.52 | <0.001 |
| 40 | 1-myristoyl-2-arachidonoyl-GPC (14:0/20:4)* | Lipid | Phosphatidylcholine (PC) | HMDB07883 | 0.72 | <0.001 |
| 41 | 1-palmitoyl-2-oleoyl-GPC (16:0/18:1) | Lipid | Phosphatidylcholine (PC) | HMDB07972 | 0.29 | <0.001 |
| 42 | 1-stearoyl-2-oleoyl-GPE (18:0/18:1) | Lipid | Phosphatidylethanolamine (PE) | HMDB08993 | 0.37 | 0.021 |
| 43 | 1-stearoyl-2-linoleoyl-GPE (18:0/18:2)* | Lipid | Phosphatidylethanolamine (PE) | HMDB08994 | 0.30 | 0.015 |
| 44 | 1-stearoyl-2-arachidonoyl-GPE (18:0/20:4) | Lipid | Phosphatidylethanolamine (PE) | HMDB09003 | 0.36 | 0.004 |
| 45 | 1-palmitoyl-2-arachidonoyl-GPE (16:0/20:4)* | Lipid | Phosphatidylethanolamine (PE) | HMDB05323 | 0.41 | 0.002 |
| 46 | 1-palmitoyl-2-docosahexaenoyl-GPE (16:0/22:6)* | Lipid | Phosphatidylethanolamine (PE) | HMDB05324 | 0.42 | 0.003 |
| 47 | 1-stearoyl-2-docosahexaenoyl-GPE (18:0/22:6)* | Lipid | Phosphatidylethanolamine (PE) | HMDB05334 | 0.32 | 0.009 |
| 48 | 1-palmitoyl-2-oleoyl-GPE (16:0/18:1) | Lipid | Phosphatidylethanolamine (PE) | HMDB05320 | 0.33 | 0.044 |
| 49 | 1-stearoyl-2-arachidonoyl-GPI (18:0/20:4) | Lipid | Phosphatidylinositol (PI) | HMDB09815 | 0.20 | 0.012 |
| 50 | glycerophosphorylcholine (GPC) | Lipid | Phospholipid Metabolism | HMDB00086 | 0.20 | 0.006 |
| 51 | glycerophosphoethanolamine | Lipid | Phospholipid Metabolism | HMDB00114 | 0.19 | 0.020 |
| 52 | choline phosphate | Lipid | Phospholipid Metabolism | HMDB01565 | 0.26 | 0.044 |
| 53 | 1-(1-enyl-stearoyl)-2-oleoyl-GPE (P-18:0/18:1) | Lipid | Plasmalogen | HMDB11375 | 0.36 | 0.004 |
| 54 | 1-(1-enyl-stearoyl)-2-arachidonoyl-GPE (P-18:0/20:4)* | Lipid | Plasmalogen | HMDB05779 | 0.36 | 0.004 |
| 55 | 1-(1-enyl-palmitoyl)-2-arachidonoyl-GPE (P-16:0/20:4)* | Lipid | Plasmalogen | HMDB11352 | 0.27 | 0.021 |
| 56 | 1-(1-enyl-palmitoyl)-2-oleoyl-GPE (P-16:0/18:1)* | Lipid | Plasmalogen | HMDB11342 | 0.25 | 0.020 |
| 57 | 1-(1-enyl-palmitoyl)-2-arachidonoyl-GPC (P-16:0/20:4)* | Lipid | Plasmalogen | HMDB11220 | 0.23 | 0.001 |
| 58 | docosahexaenoate (DHA; 22:6n3) | Lipid | Polyunsaturated Fatty Acid (n3 and n6) | HMDB02183 | 0.31 | 0.044 |
| 59 | stearidonate (18:4n3) | Lipid | Polyunsaturated Fatty Acid (n3 and n6) | HMDB06547 | 0.45 | 0.040 |
| 60 | docosapentaenoate (n6 DPA; 22:5n6) | Lipid | Polyunsaturated Fatty Acid (n3 and n6) | HMDB01976 | 0.62 | 0.016 |
| 61 | dihomo-linolenate (20:3n3 or n6) | Lipid | Polyunsaturated Fatty Acid (n3 and n6) | HMDB02925 | 0.28 | 0.047 |
| 62 | arachidonate (20:4n6) | Lipid | Polyunsaturated Fatty Acid (n3 and n6) | HMDB01043 | 0.38 | 0.003 |
| 63 | sphingomyelin (d18:1/14:0, d16:1/16:0)* | Lipid | Sphingomyelins | HMDB12097 | 0.26 | 0.001 |
| 64 | sphingomyelin (d18:2/16:0, d18:1/16:1)* | Lipid | Sphingomyelins | NA | 0.17 | 0.004 |
| 65 | sphingomyelin (d18:2/14:0, d18:1/14:1)* | Lipid | Sphingomyelins | NA | 0.24 | 0.010 |
| 66 | sphingomyelin (d18:1/20:0, d16:1/22:0)* | Lipid | Sphingomyelins | HMDB12102 | 0.18 | 0.014 |
| 67 | sphingomyelin (d18:2/18:1)* | Lipid | Sphingomyelins | NA | 0.21 | 0.010 |
| 68 | cholesterol | Lipid | Sterol | HMDB00067 | 0.20 | <0.001 |
| 69 | p-hydroxybenzaldehyde | Xenobiotics | Benzoate Metabolism | HMDB11718 | 0.48 | <0.001 |
| 70 | perfluorooctanesulfonate (PFOS) | Xenobiotics | Chemical | HMDB59586 | 0.47 | 0.010 |
| 71 | ergothioneine | Xenobiotics | Food Component/Plant | HMDB03045 | 0.40 | 0.004 |
| 72 | theophylline | Xenobiotics | Xanthine Metabolism | HMDB01889 | 1.48 | 0.015 |
| 73 | 1,3-dimethylurate | Xenobiotics | Xanthine Metabolism | HMDB01857 | 1.41 | 0.012 |
| 74 | 1,3,7-trimethylurate | Xenobiotics | Xanthine Metabolism | HMDB02123 | 1.01 | 0.015 |
| 75 | 1,7-dimethylurate | Xenobiotics | Xanthine Metabolism | HMDB11103 | 1.28 |  |

1. ANCOVA was used for the identification of metabolites after adjusting for age, educational level, physical activity, BMI, vitamin A intake, dyslipidemia medication, current smoking and current alcohol drinking. The false discovery rate was used for p-value adjustment.
2. NA, not available; HMDB, Human Metabolon Database; GPI, glycosylphosphatidylinositol; GPE, glycerophosphatidylethanolamine.

**Table S4 Significant metabolites in high- and low-retinol groups among women.**

| No. | Biochemical | Super pathway | Sub pathway group | HMDB ID | β | P-adjusted |
| --- | --- | --- | --- | --- | --- | --- |
| 1 | creatinine | Amino Acid | Creatine Metabolism | HMDB00562 | 0.13 | 0.037 |
| 2 | 3-methylglutarylcarnitine (2) | Amino Acid | Leucine, Isoleucine and Valine Metabolism | HMDB00552 | 1.04 | 0.024 |
| 3 | N-acetyltaurine | Amino Acid | Methionine, Cysteine, SAM and Taurine Metabolism | NA | 0.28 | 0.030 |
| 4 | indolelactate | Amino Acid | Tryptophan Metabolism | HMDB00671 | 0.33 | 0.018 |
| 5 | N-acetyltryptophan | Amino Acid | Tryptophan Metabolism | HMDB13713 | 0.44 | 0.022 |
| 6 | 6-bromotryptophan | Amino Acid | Tryptophan Metabolism | NA | 0.37 | 0.021 |
| 7 | pro-hydroxy-pro | Amino Acid | Urea cycle; Arginine and Proline Metabolism | HMDB06695 | 0.33 | 0.029 |
| 8 | 1-methylnicotinamide | Cofactors and Vitamins | Nicotinate and Nicotinamide Metabolism | HMDB00699 | 0.56 | 0.030 |
| 9 | dehydroisoandrosterone sulfate (DHEA-S) | Lipid | Androgenic Steroids | HMDB01032 | -0.40 | 0.020 |
| 10 | androstenediol (3beta,17beta) disulfate (1) | Lipid | Androgenic Steroids | HMDB03818 | 0.49 | 0.020 |
| 11 | androstenediol (3beta,17beta) monosulfate (1) | Lipid | Androgenic Steroids | HMDB03818 | 0.74 | 0.028 |
| 12 | androstenediol (3beta,17beta) monosulfate (2) | Lipid | Androgenic Steroids | NA | 0.50 | 0.023 |
| 13 | androsterone glucuronide | Lipid | Androgenic Steroids | HMDB02829 | 0.86 | 0.028 |
| 14 | etiocholanolone glucuronide | Lipid | Androgenic Steroids | HMDB04484 | 0.94 | 0.021 |
| 15 | 5alpha-androstan-3alpha,17beta-diol monosulfate (2) | Lipid | Androgenic Steroids | NA | 0.81 | 0.031 |
| 16 | 3-carboxy-4-methyl-5-pentyl-2-furanpropionate (3-CMPFP)** | Lipid | Fatty Acid, Dicarboxylate | NA | 0.59 | 0.048 |
| 17 | 1-palmitoyl-GPC (16:0) | Lipid | Lysophospholipid | HMDB10382 | 0.20 | 0.019 |
| 18 | 1-stearoyl-GPE (18:0) | Lipid | Lysophospholipid | HMDB11130 | 0.23 | 0.045 |
| 19 | 1-stearoyl-2-arachidonoyl-GPC (18:0/20:4) | Lipid | Phosphatidylcholine (PC) | HMDB08048 | 0.23 | 0.030 |
| 20 | 1-palmitoyl-2-arachidonoyl-GPC (16:0/20:4n6) | Lipid | Phosphatidylcholine (PC) | HMDB07982 | 0.22 | 0.025 |
| 21 | 1-linoleoyl-2-arachidonoyl-GPC (18:2/20:4n6)* | Lipid | Phosphatidylcholine (PC) | HMDB08147 | 0.34 | 0.019 |
| 22 | 1-myristoyl-2-arachidonoyl-GPC (14:0/20:4)* | Lipid | Phosphatidylcholine (PC) | HMDB07883 | 0.51 | 0.021 |
| 23 | 1-stearoyl-2-arachidonoyl-GPE (18:0/20:4) | Lipid | Phosphatidylethanolamine (PE) | HMDB09003 | 0.31 | 0.030 |
| 24 | 21-hydroxypregnenolone disulfate | Lipid | Pregnenolone Steroids | NA | 0.45 | 0.044 |
| 25 | pregnenediol sulfate (C21H34O5S)* | Lipid | Pregnenolone Steroids | NA | -0.38 | 0.046 |
| 26 | sphingomyelin (d18:1/21:0, d17:1/22:0, d16:1/23:0)* | Lipid | Sphingomyelins | NA | 0.24 | 0.047 |
| 27 | urate | Nucleotide | Purine Metabolism, (Hypo)Xanthine/Inosine containing | HMDB00289 | 0.21 | 0.046 |
| 28 | isoleucylglycine | Peptide | Dipeptide | HMDB28907 | 0.57 | 0.030 |
| 29 | gamma-glutamylvaline | Peptide | Gamma-glutamyl Amino Acid | HMDB11172 | 0.27 | 0.022 |
| 30 | 3-formylindole | Xenobiotics | Food Component/Plant | HMDB29737 | 0.22 | 0.031 |

1. ANCOVA was used for the selection of metabolites after adjustment for age, educational level, physical activity, BMI, vitamin A intake, dyslipidemia medication, current smoking, current alcohol drinking, and menopausal status. The false discovery rate was used for p-value adjustment.
2. NA, not available; HMDB, Human Metabolon Database; SAM, S-adenosylmethionine; GPE, glycerophosphatidylethanolamine; GPI. glycosylphosphatidylinositol.

**Table S5 Pathway analysis results in men.**

| Pathway | Total Compounds | Hits | Raw p | -log10(p) | Holm adjust | FDR | Impact |
| --- | --- | --- | --- | --- | --- | --- | --- |
| Glycerophospholipid metabolism | 36 | 6 | 0.00 | 6.44 | 0.00 | 0.00 | 0.32 |
| Arachidonic acid metabolism | 36 | 2 | 0.00 | 9.28 | 0.00 | 0.00 | 0.31 |
| Primary bile acid biosynthesis | 46 | 1 | 0.00 | 5.29 | 0.00 | 0.00 | 0.05 |
| Steroid hormone biosynthesis | 85 | 4 | 0.00 | 9.78 | 0.00 | 0.00 | 0.05 |
| Steroid biosynthesis | 42 | 1 | 0.00 | 5.29 | 0.00 | 0.00 | 0.03 |
| Valine, leucine and isoleucine degradation | 40 | 1 | 0.00 | 2.51 | 0.01 | 0.00 | 0.01 |
| Glycosylphosphatidylinositol (GPI)-anchor biosynthesis | 14 | 1 | 0.00 | 3.39 | 0.00 | 0.00 | 0.00 |
| Linoleic acid metabolism | 5 | 1 | 0.00 | 8.29 | 0.00 | 0.00 | 0.00 |
| Biosynthesis of unsaturated fatty acids | 36 | 3 | 0.00 | 4.95 | 0.00 | 0.00 | 0.00 |
| alpha-Linolenic acid metabolism | 13 | 2 | 0.00 | 4.50 | 0.00 | 0.00 | 0.00 |
| Ether lipid metabolism | 20 | 2 | 0.00 | 3.43 | 0.00 | 0.00 | 0.00 |
| Fatty acid degradation | 39 | 1 | 0.00 | 3.12 | 0.00 | 0.00 | 0.00 |
| Sphingolipid metabolism | 21 | 1 | 0.00 | 2.76 | 0.01 | 0.00 | 0.00 |
| Valine, leucine and isoleucine biosynthesis | 8 | 1 | 0.00 | 2.51 | 0.01 | 0.00 | 0.00 |
| Caffeine metabolism | 10 | 1 | 0.01 | 2.15 | 0.01 | 0.01 | 0.00 |

FDR, false discovery rate.

**Table S6 Pathway analysis results in women.**

|  | Total Compound | Hits | Raw p | -log10(p) | Holm adjust | FDR | Impact |
| --- | --- | --- | --- | --- | --- | --- | --- |
| Glycerophospholipid metabolism | 36 | 3 | 0.00 | 7.81 | 0.00 | 0.00 | 0.22 |
| Nicotinate and nicotinamide metabolism | 15 | 1 | 0.02 | 1.69 | 0.04 | 0.02 | 0.14 |
| Steroid hormone biosynthesis | 85 | 4 | 0.05 | 1.27 | 0.05 | 0.05 | 0.00 |
| Glycosylphosphatidylinositol (GPI)-anchor biosynthesis | 14 | 1 | 0.00 | 2.67 | 0.01 | 0.00 | 0.00 |
| Purine metabolism | 65 | 1 | 0.00 | 4.66 | 0.00 | 0.00 | 0.00 |
| Arachidonic acid metabolism | 36 | 1 | 0.00 | 4.22 | 0.00 | 0.00 | 0.00 |
| Linoleic acid metabolism | 5 | 1 | 0.00 | 4.22 | 0.00 | 0.00 | 0.00 |
| alpha-Linolenic acid metabolism | 13 | 1 | 0.00 | 4.22 | 0.00 | 0.00 | 0.00 |

FDR, false discovery rate.
